# Adaptive hip exoskeleton control using heart rate feedback reduces oxygen cost during ecological locomotion

**DOI:** 10.1038/s41598-024-84253-y

**Published:** 2025-01-02

**Authors:** Ali Reza Manzoori, Davide Malatesta, Alexandre Mortier, Johan Garcia, Auke Ijspeert, Mohamed Bouri

**Affiliations:** 1 Biorobotics Laboratory, Institute of Bioengineering, Ecole Polytechnique Fédérale de Lausanne (EPFL), CH-1015 Lausanne, Switzerland; 2 Institute of Sport Sciences, University of Lausanne (UNIL), CH-1015 Lausanne, Switzerland; 3 Laboratory Sport, Expertise and Performance (EA 7370), French Institute of Sport (INSEP), Paris, France; 4 Translational Neural Engineering Laboratory (TNE), Institute of Bioengineering, Ecole Polytechnique Fédérale de Lausanne (EPFL), CH-1015 Lausanne, Switzerland

**Keywords:** Engineering, Metabolism

## Abstract

Despite their potential, exoskeletons have not reached widespread adoption in daily life, partly due to the challenge of seamlessly adapting assistance across various tasks and environments. Task-specific designs, reliance on complex sensing and extensive data-driven training often limit the practicality of the existing control strategies. To address this challenge, we introduce an adaptive control strategy for hip exoskeletons, emphasizing minimal sensing and ease of implementation. Using only insole pressure and heart rate (HR) sensing, the controller modulates assistance across various locomotor tasks. We evaluated this strategy with twelve able-bodied participants in a real-world scenario including level walking, stairs, and inclines. The controller successfully adapted assistance timing and amplitude to different activities. This resulted in effort intensity reductions (measured by oxygen uptake) of up to 12.6% compared to walking with no exoskeleton, and up to 25.5% compared to walking with the exoskeleton in zero-torque mode. Cardiodynamic response of HR, although delayed, proved sufficient for adaptation in tasks lasting longer than around 45 s, and delay-induced limitations primarily affected brief bouts of abrupt change in intensity. However, we found discernible patterns in HR shortly after the onset of such changes that can be exploited to improve responsiveness. Our findings underscore the potential of HR as a promising measure of user effort intensity, encouraging future research to explore its integration into advanced adaptive algorithms.

## Introduction

With major advances in robotics technology at the beginning of the century, exoskeletons were predicted to become widespread by as early as 2024^1^. This vision has been realized to some extent in rehabilitation training and clinical applications, but using these devices at home and in the community remains limited^2^. Several factors at different levels contribute to this limited adoption^3,4^. When it comes to their use in uncontrolled environments and daily life in particular, a key challenge is the versatility of assistance across different locomotor tasks and environments. The vast majority of control strategies for assistive exoskeletons and exosuits in the literature target specific locomotor tasks (mostly level-ground walking), and are tested under steady-state conditions in laboratories. Locomotion in daily life, in contrast, involves navigating different terrains with variable speed and cadence^5^, mostly in short activity bouts interspersed with transitions^6^. Control strategies capable of seamlessly adapting to the varied real-world locomotion are therefore essential for extending exoskeleton functionality beyond the laboratory, making them more viable for everyday use. In the last few years, novel approaches based on either purely data-driven methods such as deep learning^7^and reinforcement learning^8^, or model-guided data-driven methods such as the extended Kalman filter^9^ have enabled addressing more realistic scenarios, including walking with variations of speed and ground inclination, stair negotiation and running, in a more unified way.

Focusing controller development on specific locomotor tasks in steady state has allowed researchers to tailor and optimize the assistance according to the specific features of each task, and impressive results have been achieved with such controllers^10^. The ubiquity of task-specific controllers has triggered a large body of research on terrain^11^ or activity^12 ^detection algorithms that allow switching between controllers or adjusting their parameters to achieve better adaptivity. However, developing robust detection algorithms generally requires large amounts of data and training, covering various tasks and/or environments. Even for the same types of activity, algorithms developed based on data collected in laboratories may show reduced performance in real environments^13^. In addition, this approach often necessitates a combination of different sensing modalities, which can complicate the design and structure of the devices and controllers, resulting in an increased risk of software and hardware failures. Furthermore, discrete transitions in modes or parameters should be carefully considered to prevent non-smooth behavior.

Another approach is to use task-invariant control strategies, thus removing the need for detection of locomotor activity or its features. Such strategies map the sensory signals measured from the human-exoskeleton system to assistive action in an inherently adaptive manner. One class of methods is based on mechanical models of the human-exoskeleton system, in which the assistive action is designed to shape the closed-loop system’s dynamics. This can be used to decrease the effective weight^14^ or inertia^15^ perceived by the user, thereby reducing their physical effort intensity. In a different approach, simplified neuromuscular models inspired by the peripheral nervous system and its reflex loops are used to map the sensory inputs to torques^16^. These methods have demonstrated adaptive behavior at different walking speeds^17^, but they have not been studied extensively in different locomotor tasks. Methods based on heuristic mappings between the sensory signals and the assistive action are a simpler alternative^18,19^, in which an ad-hoc custom function defines the mapping. Heuristic methods tend to be simpler in terms of formulation and implementation, but finding appropriate functions with adaptive behavior across various locomotor tasks is challenging. Lastly, an intuitive task-invariant approach is to quantitatively estimate the user’s instantaneous action, and generate the assistive action as a function of it. This can be in the form of proportional myoelectric control^20 ^in which muscle activations are mapped to assistive torques, or proportional joint moment control^7,21^ where the assistive torques are determined by the estimated biological joint moments.

A similar approach for improving adaptivity involves using physiological, kinematic and/or kinetic signals to estimate the overall physical effort rather than at the level of individual joints or muscles, and adapting the assistance accordingly. The most established example demonstrating the potential of this principle is human-in-the-loop optimization of assistance based on metabolic rate measurements^22^. However, this method is also limited to laboratory settings since the existing metabolic rate measurement systems cannot be integrated into daily use. Various alternative signals that are measurable with less obtrusive wearable sensors have been proposed^23,24^, and even successful integrations of such signals into control strategies have been reported^25^. One of the candidate signals is the heart rate (HR)^23^, which can be accurately measured with low-cost, lightweight, and unobtrusive sensors^26^. The popularity of HR sensors among everyday users and athletes^27^ highlights their accessibility and ease of use. Despite its salience as a surrogate for effort intensity and its clear practical advantages, HR has been largely overlooked in the exoskeleton literature as an input for assistance adaptation.

**Fig. 1:**
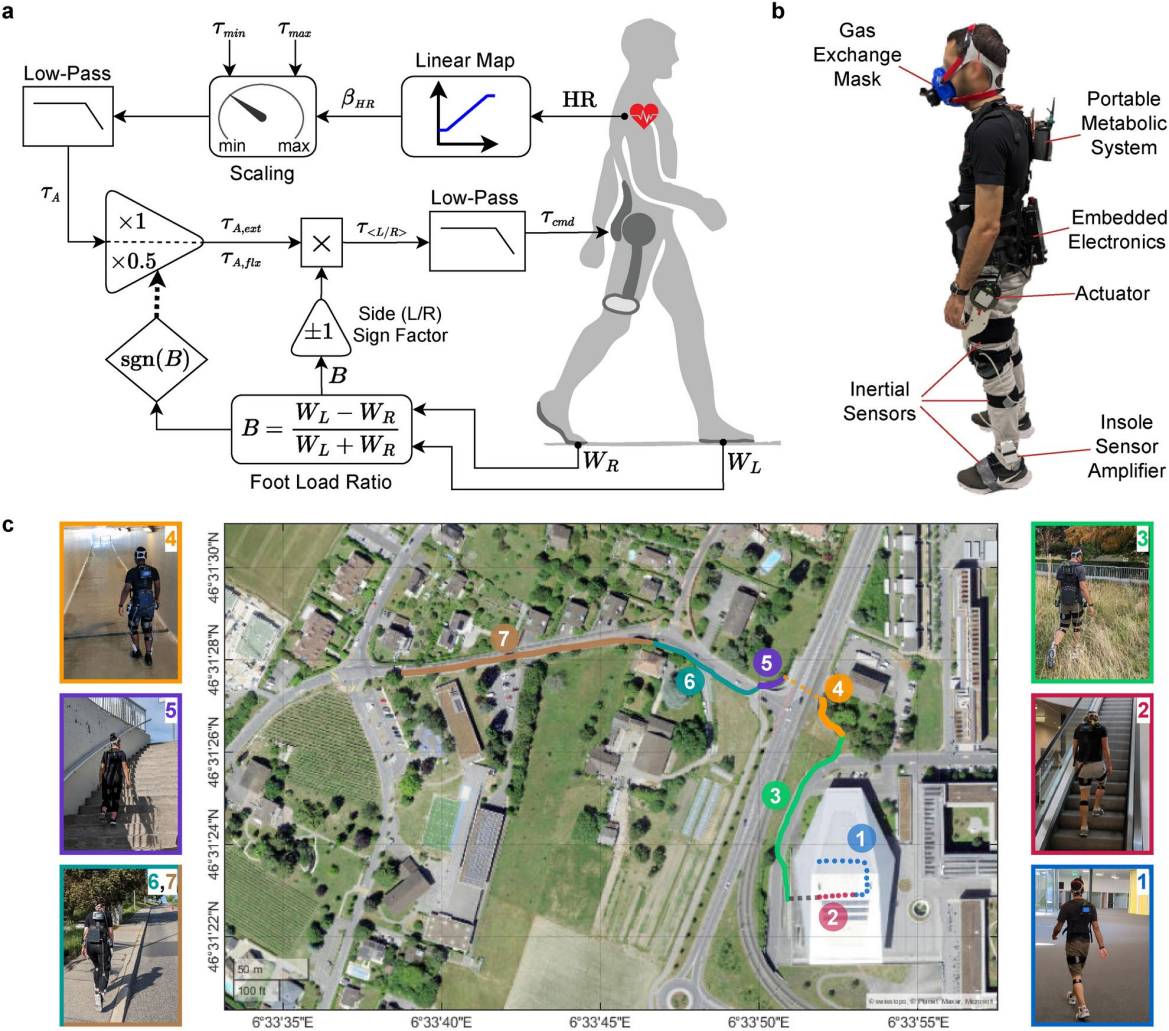
Methods and setup. **(a)** The schematic structure of the Simple Reflex Controller with Heart Rate Adaptation (SRC-HrA). **(b)** Testing and measurement setup, including the e-Walk V2 exoskeleton, inertial motion capture, and metabolic measurement systems. **(c)** The testing route and its subsections: (1) Indoor flat, (2) Escalator (simulated stair ascent), (3) Outdoor init., (4) Underpass, (5) Stairs, (6) Incline init., (7) Incline cont. Dashed line is used for the indoor sections and solid line for the outdoor parts. The map view was generated using MATLAB R2022b^28^.

In a previous study^29^, we presented a control strategy for hip exoskeletons based on the simple principle of assisting hip extension during stance and hip flexion during swing. A mapping from the ground reaction forces on the feet to the assistive torques was proposed to define the shape and timing of assistance, and the amplitude was determined by a scaling parameter that was manually tuned according to the target activity. Here, we expand this controller by using HR feedback to adapt the assistive torque amplitude to the specific intensity of the locomotor task (Fig. 1a). The expanded strategy, named Simple Reflex Controller with Heart rate Adaptation (SRC-HrA), has a simple structure with very few parameters, and requires minimal sensor data (only approximate vertical ground reaction forces and HR). We hypothesized that this strategy would appropriately adapt the assistance amplitude to different locomotor tasks without any task-specific tuning. We defined appropriate adaptation as an amplitude adjustment in proportion to the hip joint torque amplitude needed for each type of locomotor activity. The adaptation quality was thus evaluated by the correlation coefficient between biological and assistive torque amplitudes across tasks, with a strongly positive correlation (Pearson’s $r> 0.7$) indicating good adaptation. We also investigated the general feasibility of HR as a real-time feedback of user effort intensity by studying its response to changes in locomotor task intensity.

We implemented SRC-HrA on an autonomous hip exoskeleton (Fig. 1b) and evaluated its performance with 12 able-bodied participants in a real-world scenario (Fig. 1c and Supplementary Video). The testing scenario consisted of a mixed-terrain route with 7 sections, starting with an indoor part and continuing outdoors, for a total duration of 10 min. The indoor part began with level-ground walking (“Indoor flat”), followed by simulated stair climbing on a descending escalator (“Escalator”), each lasting 2 min. The scenario continued outdoors for 6 min, beginning along a mostly flat urban route (“Outdoor initial”, shortened to “Outdoor init.” for brevity) extending to the entrance ramp of a pedestrian underpass (“Underpass”). A flight of stairs at the end of the underpass (“Stairs”) led to an uphill sidewalk, with an initial section of gradually increasing inclination (“Incline initial”, abbreviated as “Incline init.”), and continuing with a near-constant inclination until the end (“Incline continuation”, abbreviated as “Incline cont.”). For more details about the route, see Methods, Supplementary Methods, and the Supplementary Video. We tested the scenario under three conditions: without the exoskeleton (“No Exo”), wearing the exoskeleton in zero-torque mode with only minimal resistance from actuators’ internal friction and inertia (“Exo Off”), and assisted with SRC-HrA (“Exo On”). Each participant followed their preferred walking pace pattern, with natural adjustments between and within sections. This pattern was established during a familiarization session along the same route with exoskeleton assistance, and was consistently maintained across all three conditions. We analyzed the controller’s adaptive behavior across terrains by examining changes in assistance timing and amplitude. To validate the amplitude adaptation, we compared the resulting assistance amplitude in each section to average biological hip torque amplitude for the corresponding locomotor task. The quality of assistance was further assessed in terms of its impact on the energetics, kinematics and spatiotemporal parameters of gait. We also analyzed the response timing characteristics of the HR signal to determine its suitability as a real-time measure of effort intensity for assistance adaptation, independently of the proposed control strategy.

## Results

### Assistance adaptivity

**Fig. 2:**
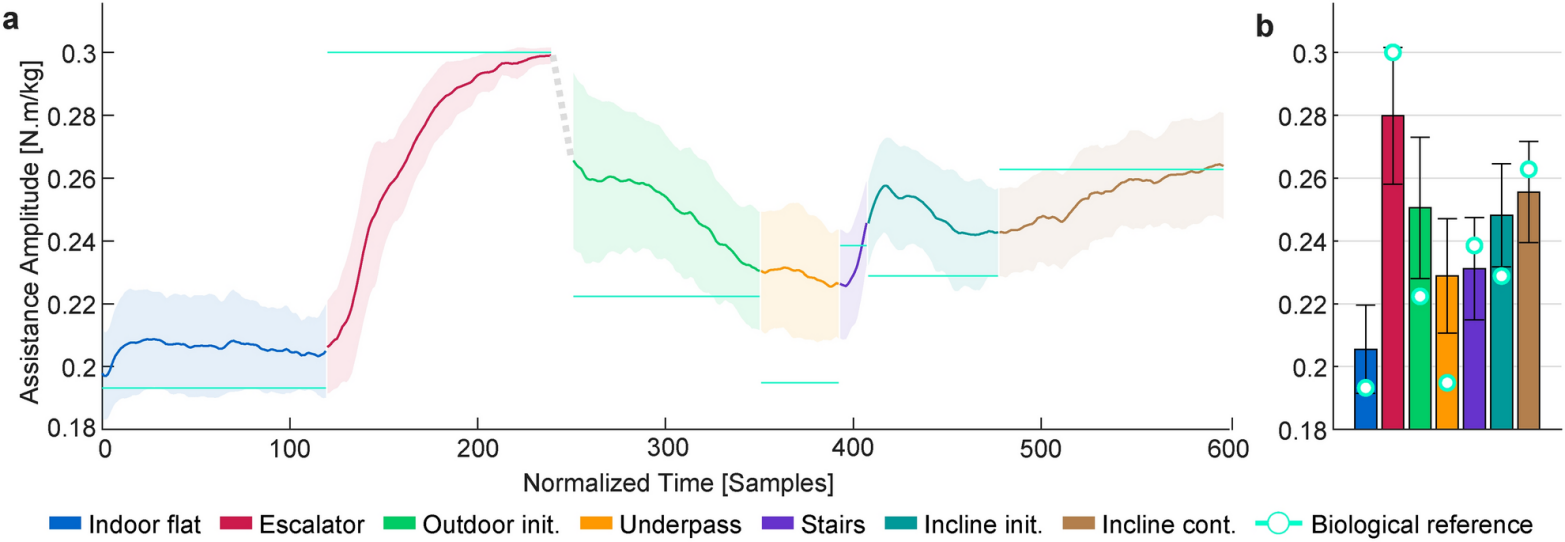
Assistance amplitude adaptation across different sections. **(a)** The evolution of the amplitude over time, averaged over participants. The dashed gray line represents the excluded transition period between the indoor and outdoor sections. The shaded area marks the standard deviation, and the horizontal lines show the biological reference values for each section. The values for individual participants were resampled to ensure a uniform number of samples in each section before averaging. **(b) **The average amplitude of assistance for each section. The error bars denote the standard deviation of participant means, and the circular markers show the biological reference values. The biological reference values are based on hip torque amplitudes reported in the literature for matched locomotor tasks^30,31^, averaged across several individuals.

The assistance amplitude varied continuously with changing physical intensity levels along the testing route, as shown in Fig. 2a. The average amplitudes displayed noticeable differences among the sections (Fig. 2b), showing a strong positive correlation with the range-mapped biological reference values associated with each section (Pearson’s $r(5) = 0.879, p=0.009$). These reference values were obtained by scaling the average biological hip torque amplitudes for corresponding locomotor tasks to fall within the controller’s range of assistance amplitudes. The highest assistance amplitudes, occurring in Escalator and Incline cont., were ${0.280\pm 0.022}\hbox { N} \cdot \hbox {m/kg}$ and ${0.255\pm 0.016}$ $\hbox {N} \cdot \hbox {m/kg}$ respectively. The lowest amplitude of ${0.205\pm 0.014}$ $\hbox {N} \cdot \hbox {m/kg}$ corresponded to Indoor flat.

**Fig. 3:**
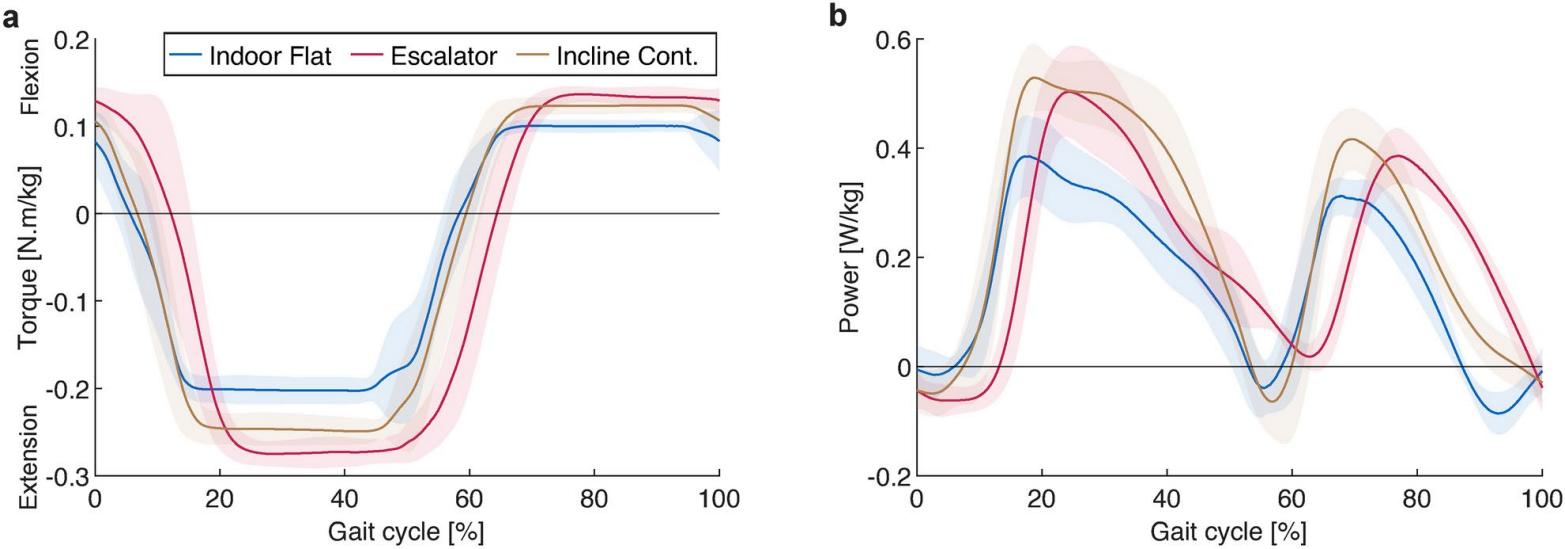
Average assistance profiles for walking on level and inclined ground and ascending stairs (on the escalator). **(a)** Exoskeleton torque commands, **(b)** Exoskeleton mechanical powers. The shaded areas mark the standard deviation.

In addition to the changes in amplitude driven by HR, the timing of assistance also showed variations across terrains, driven by variations in the temporal evolution of the foot loads. The average torque and power profiles for three terrain types (level ground, upward slope, and stairs) are illustrated in Figs. 3a and 3b. Compared to level ground, the extension assistance phase showed a slightly delayed onset in inclined walking (${\sim }1.2\%$ of the gait cycle), and a more substantial delay in stair ascent (${\sim }6.6\%$). The relative durations of extension and flexion assistance remained similar among the profiles (${\sim }53\%$ of the gait cycle for extension). The exoskeleton predominantly delivered positive mechanical power, with some brief negative power periods during double support. The power profiles had two distinct peaks around mid-stance and mid-swing, the timings of which displayed similar relative delays to the torque profiles, increasing from level to inclined ground to stairs.

### Heart rate response

The HR response is known to be dynamically delayed, which causes lags in the adaptation of assistance and may lead to carry-over effects when transitioning from one section to the next. To quantify this behavior, we characterized the responsiveness of HR to abrupt changes in activity (i.e., from walking to stair ascent and vice versa). We used two metrics: response time (the time between the change in activity and the onset of detectable change in HR—see Methods for details), and time constant (the time required for reaching $\sim$63.2% of the total change in HR). The values are reported in Table 1. The initial response to abrupt changes in intensity was relatively rapid (occurring within ${\sim }{5}\hbox { s}$ on average, and no later than 33 s) and even preceded the change in activity by up to 20 s in the initiation phase of the Escalator and Stairs sections for some participants (see also Supplementary Fig. S4). Approaching a near-steady-state value (characterized by a coefficient of variation below 10%) took place more slowly, with a time constant of around 44 s.

**Table 1:** HR response temporal metrics. Response times were calculated for initiation and termination of ascent on the escalator, and initiation of stair ascent. Time constant was only calculated for the start of escalator ascent. Values are reported as $\textrm{mean}\pm \mathrm {s.d.}\,\mathrm {[min.,max.]}$. The sample sizes for all of the Escalator response metrics in Exo On and also Escalator (Termination) and Stair (Initiation) response times in No Exo are $n=11$ due to unusable data for one participant.

Condition	Response Time [s]			Time Constant [s]
	Escalator (Initiation)	Escalator (Termination)	Stairs (Initiation)	Escalator (Initiation)
No Exo	$-3.25\pm 10.75\, [-20,16]$	$5.0\pm 9.64\, [0,33]$	$0.91\pm 3.5\, [-7,5]$	$45.17\pm 12.33\, [23,63]$
Exo On	$-5.27\pm 7.50\, [-19,8]$	$2.36\pm 2.69\, [0,8]$	$1.92\pm 4.81\, [-10,9]$	$43.82\pm 13.61\, [23,67]$

### Effects on energetics of gait

**Fig. 4:**
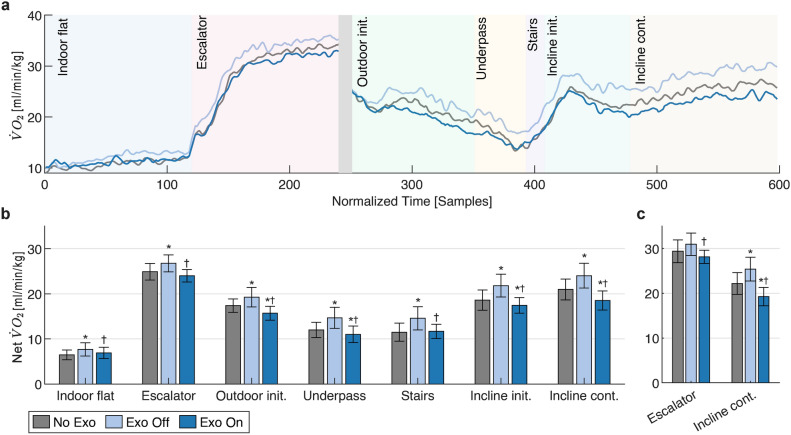
Average gross and net (gross minus resting) oxygen uptake ($\dot{\textrm{V}O_2}$) among conditions. **(a)** Evolution of gross $\dot{\textrm{V}O_2}$ over the entire experiment. The values for individual participants were resampled to enforce a uniform number of samples in each section, and then a moving average with a window size of 5 was applied. Background colors demarcate the sections, and the gray area marks the excluded transition period between the indoor and outdoor sections. **(b)** Net $\dot{\textrm{V}O_2}$ averaged over the entire duration of each section. **(c)** Net $\dot{\textrm{V}O_2}$ averaged over the last 30 s of sections with sustained activity. Error bars mark the standard deviation. Significant differences ($p<0.05$) with respect to No Exo and Exo Off are marked with ^*^ and $^{\dagger }$, respectively.

Exoskeleton assistance led to reduced energy consumption (based on net oxygen uptake, $\dot{\textrm{V}O_2}$) compared to Exo Off (over the entire route) and No Exo (in all sections except Indoor flat, Escalator and Stairs), as shown in Fig. 4a. Similar patterns were observed in section-level averages as well (Fig. 4b). Compared to No Exo, net $\dot{\textrm{V}O_2}$ showed significant increases of 8.0% (Escalator, Cohen’s $d=1.315$, $p=0.002$) to 22.7% (Indoor flat, $d=0.922$, $p=0.005$) in Exo Off on average. Assistance significantly reduced the values by 10.0% (Escalator, $d = -1.701$, $p<0.001$) to 25.5% (Underpass, $d=-2.394$, $p<0.001$) compared to Exo Off. Between Exo On and No Exo, significant reductions of 6.9% (Incline init., $d=-0.644$, $p=0.019$) to 11.8% (Incline cont., $d=-2.741$, $p<0.001$) were observed in net $\dot{\textrm{V}O_2}$; on the other hand, there were non-significant reductions of 2.8% in Escalator ($d=-0.389$, $p=0.151$) and 0.8% (Stairs, $d = -0.047$, $p=0.898$), and a non-significant increase of 5.3% in Indoor Flat ($d=0.321$, $p=0.386$). The differences among the conditions became more pronounced toward the end of the experiment, as can be seen in Fig. 4a. For the two sections with largely constant intensity for about 2 min (i.e., Escalator and Incline cont.) in which $\dot{\textrm{V}O_2}$ appeared to approach a nearly steady state towards the end (see Methods and Supplementary Methods), we also compared the results for the last 30 s, shown in Fig. 4c. During this period, assistance reduced net $\dot{\textrm{V}O_2}$ compared to No Exo by 12.6% in Incline cont. ($d=-1.883$, $p < 0.001$), down from a 15.2% ($d=2.772$, $p <0.001$) increase in Exo Off. Furthermore, there was an apparent reduction of 4.4% in Escalator ($d=-0.576$, $p = 0.073$), down from an apparent 4.0% ($d=0.807$, $p = 0.073$) increase in Exo Off, none of which reached statistical significance.

### Effects on kinematics and spatiotemporal parameters

**Fig. 5:**
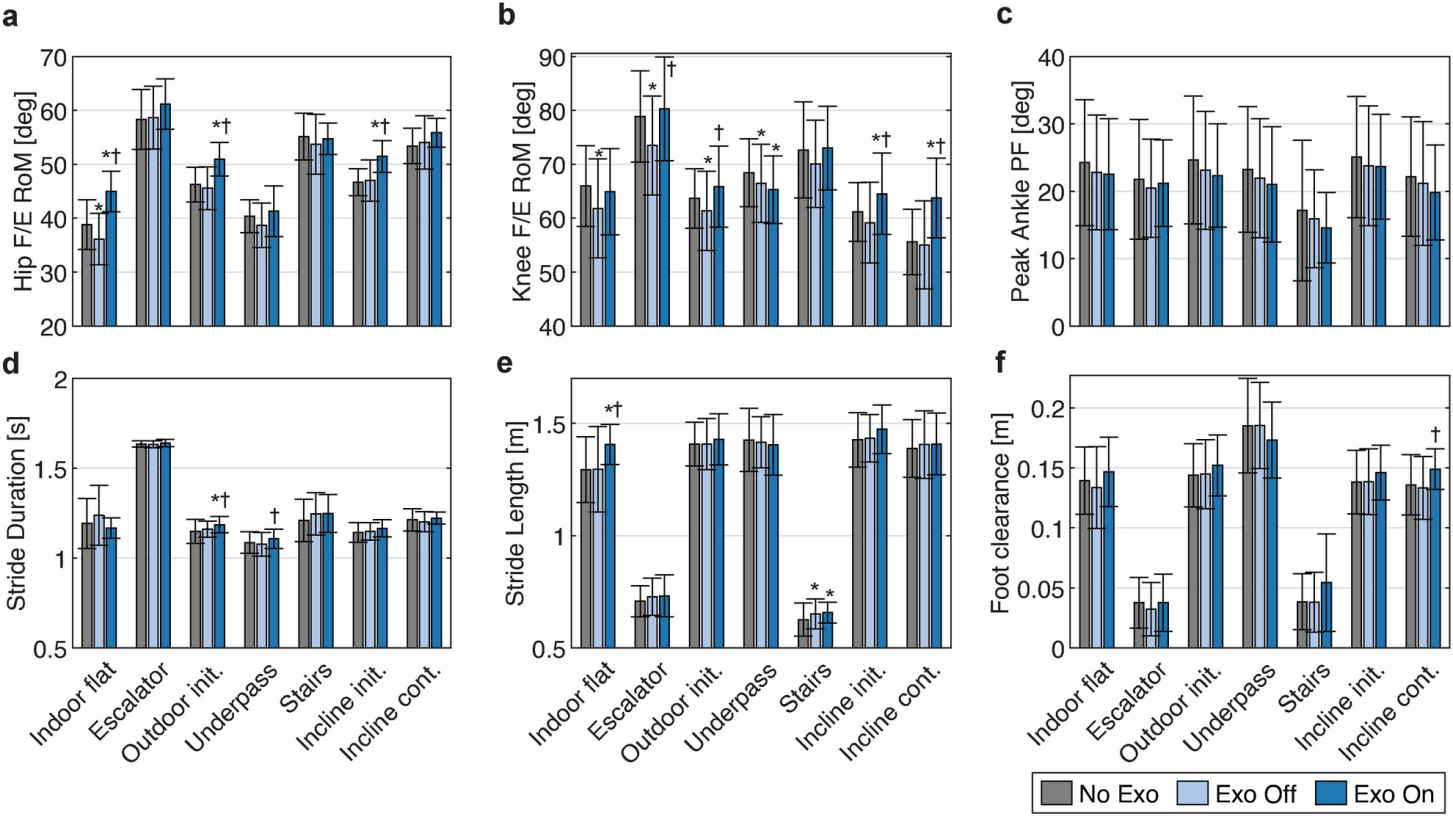
Comparison of selected kinematic and spatiotemporal parameters among conditions. **(a)** Range of motion (RoM) of hip flexion/extension. **(b)** RoM of the knee flexion/extension. **(c)** Peak ankle plantarflexion. **(d)** Stride duration. **(e)** Stride length. **(f)** Maximum foot clearance. Error bars show standard deviations. Significant differences ($p<0.05$) with respect to No Exo and Exo Off are denoted with ^*^ and $^{\dagger }$, respectively.

As a result of the assistance, we observed increases in the average ranges of motion (RoMs) of hip and knee joints in all sections except in Stairs for the hip, and Indoor flat and Underpass for the knee (Figs. 5a–b). This increase (Exo On w.r.t. No Exo) was significant for hip RoM in Indoor flat ($\Delta \mathrm {RoM_{hip}}={6.1}^{\circ }$, $p<0.001$), Outdoor init. ($\Delta \mathrm {RoM_{hip}}={4.7}^{\circ }$, $p<0.001$), and Incline init. ($\Delta \mathrm {RoM_{hip}}={4.8}^{\circ }$, $p<0.001$), and for knee RoM in Incline init. ($\Delta \mathrm {RoM_{knee}}={3.3}^{\circ }$, $p=0.007$) and Incline cont. ($\Delta \mathrm {RoM_{knee}}={8.1}^{\circ }$, $p<0.001$). The only exception was Underpass (which involved downhill walking), where the knee RoM significantly decreased ($\Delta \mathrm {RoM_{knee}}={-3.1}^{\circ }$, $p<0.001$) with assistance. On the other hand, the peak ankle plantarflexion angle had a decreasing tendency on average (Figs. 5c), although not statistically significant. The complete profiles of the average hip, knee and ankle joint angles are shown in Supplementary Figs. S5–S11. Stride durations remained similar between conditions, with differences below 0.04 s (Fig. 5d). Stride lengths only showed a notable increase of 0.11 m ($p=0.009$) between Exo On and the unassisted conditions in Indoor flat, while the difference remained below 0.05 m in all other sections (Fig. 5e). Lastly, foot clearance showed an increasing trend with assistance, which was non-significant but nevertheless consistent in all sections except Underpass (Fig. 5f).

## Discussion

Overall, a good correspondence was observed between assistance amplitudes and the required physical effort across locomotor activities. This was demonstrated by a strong correlation between the average expected biological hip torques and the resulting assistance amplitudes in each section. As shown in Fig. 2b, in four out of the seven sections, the scaled biological hip torque amplitude for the corresponding locomotor task was within one standard deviation from the mean assistance amplitude. This was not the case for Outdoor init., Underpass, and Incline init. sections, where the assistance amplitude was higher than the biological reference. These sections occurred after periods of stair ascent, and therefore the excessive assistance amplitude was likely due to the carry-over effect of the high activity intensity prior to them. Note that this effect was artificially mitigated in Outdoor init., due to the transition period from indoor to outdoor which we excluded from our analysis. These carry-over effects are unlikely to pose safety risks in partial assistance exoskeletons; but insufficient or excessive levels of assistance can cause inefficiencies, discomfort, or reduced metabolic gains, as discussed later in the section. Despite a few discrepancies in the mean assistance amplitudes compared to the biological references, the overall direction of adaptation in all sections agreed with the direction of change in the biological reference, except in the beginning of Incline init. where the increasing trend from Stairs briefly continued. In addition to the agreement with biological reference torques, the correspondence between the assistance and the expended physical effort was also reflected in the similar trend between the assistance amplitudes and $\dot{\textrm{V}O_2}$.

Delays resulting from cardiorespiratory dynamics affected both the assistance amplitude and $\dot{\textrm{V}O_2}$, as evident in their elevated values shortly after the Escalator and Stairs sections (Figs. 2a and 4a). Not surprisingly, both HR and $\dot{\textrm{V}O_2}$ showed similar responses, and our estimated time constant of ${\sim }{45}\hbox { s}$ is very close to the time constant reported for metabolic response calculated from respirometry^32^. Another consequence of these delays was that the full adaptation of assistance to sudden changes in effort required some time, although the majority of this adaptation (i.e., 63.2%) occurred within less than 1 min. Even though based on our comparative analysis with respect to biological reference torques, the assistance was adequately responsive for most sections, this delay can become consequential during short bursts of intense activity, as observed most clearly in the Stairs section. However, during such abrupt changes in the intensity of activity, the initial HR response was very rapid (below 10 s in more than 90% of the samples, and on the order of a few seconds on average, see Supplementary Fig. S4 and Table 1), and could even precede the onset of change in intensity, due to the known phenomenon of anticipatory HR response^33^. This suggests that predictive adaptation algorithms could be developed based on the early response. In line with this observation, incorporating the rate of change of such physiological signals into estimation algorithms has been previously proposed in the literature as a solution to improve responsiveness^34^. Given that around 80% of the walking bouts in daily life have been observed to last 20 s and longer^35^, such HR-driven predictive algorithms have the potential to be sufficiently responsive in most everyday scenarios. In outdoor settings (where augmentative assistance is arguably more relevant), the delay is even less concerning since the walking bouts tend to have longer durations^36^. That said, the robustness of such algorithms against false positives as a result of natural HR fluctuations must be ensured. In our analysis, we found the number of persistently increasing/decreasing HR samples to be a more reliable metric than its instantaneous time derivative, due to a lower sensitivity to short-term oscillations. On the other hand, in cases involving short ($\le {10} \hbox { s}$) bursts of effort like quick stair climbs, or continuous large variations in physical intensity such as navigating highly undulating terrain, and also for user populations suffering from moderate to severe mobility deficits whose walking bouts tend to be short and intermittent, purely HR-driven adaptation is insufficient.

Several other caveats also exist when considering HR as a surrogate for effort intensity, namely intra- and inter-personal variability, sensitivity to other factors such as emotional arousal and environmental conditions, and the non-linearity of the relationship between HR and metabolic rate beyond the moderate HR range^26,37^. For example, the notable inter-individual variability observed in HR response times underscores the challenge of creating a one-size-fits-all controller and suggests that individualized settings could enhance effectiveness. The potential impact of these factors might be mitigated through a robust control design that incorporates filtering techniques, adaptive thresholds, and brief calibrations for personalization. Furthermore, these issues are less critical when utilizing HR for coarse amplitude adjustments, as opposed to precise, fine-tuned control. Another challenge would arise if the levels of assistance are high enough to substantially offset or eliminate increases in HR. While most portable and lightweight exoskeletons cannot deliver torques of this magnitude, in such circumstances the current approach of scaling the assistance amplitude can cause amplitude fluctuations. Adopting a set-point-based regulation approach, in which the assistance amplitude is proportional to the error between the measured HR and a target value could prevent such oscillatory behavior.

Adapting the assistance amplitude to match the user’s physical effort and task demands has the potential to enhance both the alignment of the assistive torques with the user’s intention and the energy efficiency of the device compared to fixed-level strategies. While we cannot isolate the effect of HR-driven adaptation due to the lack of a fixed-amplitude control condition in this study, previous research has demonstrated the utility of task-specific adjustments. In studies using human-in-the-loop optimization of assistance for metabolic cost reduction, clear trends of increasing amplitude for more physically demanding tasks have emerged^25,38^, with task-adapted assistance providing up to 72% more reduction in metabolic cost compared to generic assistance^25^. In contrast, insufficient amplitudes relative to the demands of the task result in low metabolic gains below the perception threshold of the users^39^, thus failing to encourage adoption. On the other hand, excessive levels of assistance lead to discomfort^40^, induce unnatural movements^41^ (such as the significant increase in hip RoM observed in the Outdoor init. and Incline init. sections), and do not necessarily result in higher metabolic gains^41^. Furthermore, long-term use of high assistance may also give rise to over-reliance and deconditioning^42^. Lastly, from a practical standpoint, the increased energy consumption shortens the device’s operational time. For instance, maintaining the maximum assistance level throughout our mixed-terrain scenario would have increased the effective motor currents by approximately 20% on average (see Supplementary Methods for details), therefore reducing battery life by more than 20%.

In terms of timing and synchronization within the gait cycle, the directions of exoskeleton torques were mostly aligned with those of the participants’ movements, as evidenced by the predominantly positive mechanical powers which covered more than 80% of the gait cycle on average. The brief periods of negative power occurred around transitions between stance and swing. In walking, this included both transitions, while in stair climbing, only the swing-to-stance transition was affected. The negative powers were due to the fact that the controller assists the extension and flexion movements, respectively, after the stance and swing phases are initiated by the user. Since the hip changes its direction of movement shortly before the initiation/termination of ground contact, the exoskeleton torques briefly oppose the movement. Nonetheless, the controller showed fairly good adaptive behavior across terrains. Notably, the observed trend of increasing delay in extension assistance from level to inclined ground to stairs is in line with the relative timings observed in biological hip moments^43^. It must be noted that the current design aligns well with tasks involving positive power generation. In contrast, for tasks dominated by negative power such as downhill walking and stair descent, where the primary need is controlled deceleration, the value of active assistance is limited, and a different assistance paradigm would be more appropriate.

In addition to inherent adaptivity across terrains, the proposed control strategy demonstrated other promising features. In particular, the assistance automatically dropped nearly to zero during short periods of standstill, which is an important feature for assistance in the intermittent walking bouts of daily life. Additionally, since the input variables of the controller (i.e., the vertical ground reaction forces) were not directly affected by the control action, the risk of unstable interaction loops was eliminated. Such interactions may occur when kinematic signals of the assisted joints or segments determine the torques or forces that directly act on them, and are particularly risky at high amplitudes of assistance.

The adaptive assistance resulted in a consistent reduction of $\dot{\textrm{V}O_2}$compared to Exo Off in all sections, despite substantial variations in locomotor activities and levels of physical intensity across sections. Furthermore, the effect of assistance surpassed the considerable added cost of the exoskeleton’s mass (6 kg), resulting in net reductions of oxygen uptake compared to No Exo in all sections except Indoor flat and Stairs, though the apparent reduction in Escalator was not statistically significant. In Stairs, the duration was too short (around 15 s) for the assistance amplitude to adapt properly. In the case of Indoor flat, which served as a warm-up period, the reduction was most likely limited due to the insufficient initial re-acclimation of the participants to assistance. Notably, in our previous study^29^ the fixed-amplitude variant of this strategy yielded an average metabolic rate reduction of 8.8% in level-ground walking with lower assistive torques (versus the apparent 5.3% increase in Indoor flat in the current study). The re-acclimation hypothesis is further supported by the larger increases in stride length and hip RoM (Figs. 5a and 5e) in Indoor flat, despite the relatively low amplitude of assistance. Indeed, it is likely that the gradual acclimation of the participants had a considerable effect on the overall results as well, since the reductions in $\dot{\textrm{V}O_2}$ were most prominent toward the end, with the highest reduction of 12.6% occurring in the final 30 s of the route. Note that we recruited naïve participants who only had one brief familiarization session, giving them less than 20 min of prior exposure to the assistance. Therefore, the observed outcomes likely present a lower bound on the achievable energetic benefits, which can increase 2–3 times with more training^44^. For perspective, one of the most notable results reported in real-world testing has been a 17% reduction in the cost of transport, achieved with expert participants (8+ h of experience) using human-in-the-loop optimization of ankle assistance^25^. Moreover, this was accomplished with peak torques of around 0.75 $\hbox {N} \cdot \hbox {m/kg}$ —more than twice the values in our study.

It must be noted that direct conclusions about absolute changes in metabolic cost cannot be drawn in this study, due to methodological limitations. Firstly, because of vigorous physical exertion during the 2 min of stair ascent in the Escalator section, the respiratory exchange ratio (RER) exceeded 1.0 toward the end. This means that a greater proportion of energy production was through anaerobic metabolism, indicating that indirect calorimetry can no longer accurately estimate the metabolic rate^45^. Furthermore, use of indirect calorimetry in non-steady-state tasks is complicated and additional measures such as the inclusion of post-exercise oxygen consumption have been suggested for a more accurate estimation of metabolic rate^46^. Nevertheless, since our focus was to compare the relative effort among the three conditions in similar circumstances, these issues should not be considered important experimental limitations for our study.

Although there were few significant changes in the kinematic and temporal characteristics of gait, some trends were observed that could provide indications about the mechanisms of physical effort reduction. When assisted, the hip RoM showed significant increases at the beginning of the experiment and during periods of excessive assistance due to carry-over effects (i.e., Outdoor init. and Incline init., both succeeding a period of stair ascent). However, as the participants adapted to assistance, the increase diminished, suggesting a reduction in their hip muscle forces. The increased hip RoM also led to longer stride lengths and durations (since the gait speed was constant). In addition, the knee RoM also showed an increasing trend in Exo On, particularly in sections involving level-ground or uphill walking. This was mainly due to more extended knee angles in the stance phase, which resulted from the extension torques applied by the exoskeleton. A more extended posture at the knees would naturally lead to a reduction in the required extension torque for body weight support because of smaller moment arms. Increased knee extension in stance is known to emerge when optimizing for energy consumption in simulations^47 ^and also as an energy-saving mechanism in obese adults^48^. Furthermore, small reductions observed in peak plantarflexion angles suggest a decreased reliance on ankle push-off. Lastly, the tendency toward increased foot clearance indicates that the provided assistance may prevent falls by reducing the likelihood of tripping, which is a major cause of loss of balance^49^. This is particularly important for the older population, who faces higher risks of falls due to reduced foot clearance.

In conclusion, this study demonstrated the promise of SRC-HrA as a simple yet versatile control strategy for task-invariant assistance. Designed with real-world usability in mind, the controller could robustly operate with a minimal set of unobtrusive and low-cost wearable sensors, requiring very little person- and task-specific tuning. In testing across diverse ecological locomotor activities, the controller modulated the assistance in terms of timing (within gait cycles) and amplitude (across locomotor tasks) with reasonable responsiveness, leading to significant reductions in physical effort intensity. The biomechanical analysis showed indications that the observed reductions in physical effort were not isolated to the hip, but extended to the knee and ankle joints, suggesting a comprehensive lower-limb assistive effect. Beyond the specific findings on SRC-HrA, our results indicate the potential of HR feedback for dynamically adapting assistance across various locomotor activities encountered in daily life. Although with a linear mapping, the adaptation was insufficient during short bouts of abrupt change in physical task intensity, the HR data exhibited precursory trends that could be exploited to improve the adaptation response. Despite some limitations, the practicality of HR thanks to low-cost, unobtrusive yet accurate sensors offers a compelling trade-off. Our findings warrant further exploration of HR-driven adaptation of assistance and its integration into various control paradigms.

## Methods

### Controller

#### Base structure: Simple reflex controller

The basic structure of the SRC has been described in previous work^29^. Here, we will briefly present the structure again for completeness. The output of the controller is governed by a variable *B* defined as:

1 $$\begin{aligned} B(W_L, W_R) = \frac{W_L - W_R}{W_L + W_R} \end{aligned}$$

where $W_L$ and $W_R$ are the vertical ground reaction forces on the left and right feet, respectively. With this definition, *B* ranges from $+1$ during the single-support phase of the left leg to $-1$ during the right single-support, with the transition occurring during double support. In standstill (when $W_L \approx W_R$), the value will be close to zero. Note that due to the normalized and dimensionless nature of *B*, the vertical ground reaction forces do not need to be accurately calibrated. The assistive torques for the left ($\tau _L$) and right ($\tau _R$) sides are then calculated as:

2 $$\begin{aligned} \tau _L(B) = {\left\{ \begin{array}{ll} -B \, \tau _{A,ext} & B> 0\\ -B \, \tau _{A,flx} & B \le 0 \end{array}\right. } \end{aligned}$$

3 $$\begin{aligned} \tau _R(B) = {\left\{ \begin{array}{ll} B \, \tau _{A,ext} & B < 0\\ B \, \tau _{A,flx} & B \ge 0 \end{array}\right. } \end{aligned}$$

where $\tau _{A, ext}$ and $\tau _{A, flx}$ determine the assistance amplitude in the extension and flexion directions, respectively (positive sign indicates flexion). Thus, the controller will alternate between extension assistance during the stance phase of each leg and flexion assistance during swing. This is based on the principle that the hip joint works to propel the body forward during stance, and moves the leg forward during swing. This principle applies to forward and upward locomotion in general, regardless of the specific terrain (i.e., level and inclined ground, or stairs).

#### Adaptation based on heart rate

We used the HR signal to adapt the assistance amplitude using a linear mapping. The mapping is based on a definition similar to the percentage of the HR reserve, a common individualized metric of exercise intensity^50^, which we adapted as:

4 $$\begin{aligned} \beta _{\textrm{HR}}(t) = \frac{\textrm{HR}(t) - \mathrm {HR_{min}}}{\mathrm {HR_{max}}-\mathrm {HR_{min}}} \end{aligned}$$

where $\textrm{HR}(t)$ denotes the instantaneous value of the HR, and $\mathrm {HR_{min}}$ and $\mathrm {HR_{max}}$ are the lower and upper bounds of the operating HR range, which can be tuned to adjust adaptation sensitivity depending on the user’s HR dynamics. In this study, we set $\mathrm {HR_{min}}$ and $\mathrm {HR_{max}}$ for each individual to the minimum and maximum HR values recorded during a familiarization session in which the participant walked at their self-selected speed in a range of terrains, including the same route as in the main experiment (see Supplementary Methods for details). In the case of HR values falling outside of the $[\mathrm {HR_{min}},\mathrm {HR_{max}}]$ range, $\beta _{\textrm{HR}}$ was enforced to remain in the [0, 1] interval. The assistance amplitude was then calculated as:

5 $$\begin{aligned} \tau _A = \tau _{\text {min}} + \beta _{\text {HR}} \cdot \left( \tau _{\text {max}}-\tau _{\text {min}} \right) \end{aligned}$$

where $\tau _{\text {min}}$ and $\tau _{\text {max}}$ are the lower and upper bounds of assistance amplitude. For this study, we set $\tau _{\text {min}} = {0.19} \hbox { N} \cdot \hbox {m/kg}$, which was determined experimentally based on comfort for naïve users during level-ground walking, and $\tau _{\text {max}} = {0.3} \hbox { N} \cdot \hbox {m/kg}$, which was imposed by the torque capacity of the exoskeleton based on the maximum user body mass. For extension assistance ($\tau _{A, ext}$), the full amplitude was used, but for flexion assistance ($\tau _{A, flx}$), only 50% of the amplitude was applied, in accordance with the range of flexion-to-extension torque ratios in optimal assistance profiles found for walking on level to mildly inclined ground^38^. To prevent transient variations in HR, which have frequencies around 0.1 Hz^51^, from causing fluctuations in assistance, $\tau _A$ was filtered using a first-order low-pass filter with a cut-off frequency of 0.16 Hz.

### Experimental setup

#### Equipment

An autonomous hip exoskeleton (e-Walk V2) was used to implement and test the controller. The exoskeleton’s backdrivable actuators can provide torques of up to 35 $\hbox {N} \cdot \hbox {m}$ in the sagittal plane, with passive degrees of freedom around the other axes. For approximate vertical ground reaction force measurement ($W_L$ and $W_R$ in Eq. 1), we directly used the outputs of insole force-sensitive resistors, which are proportional to the vertical ground reaction forces on the feet. These sensors were connected via cables to the exoskeleton’s embedded computer. The total mass of the device, including batteries and electronics, is 6 kg. More details about the device are provided in Supplementary Methods.

HR was measured using an ECG chest strap (H10, Polar Electro Oy, Finland) at 1 Hz and transmitted to the exoskeleton’s embedded computer via Bluetooth. Breath-by-breath oxygen uptake ($\dot{\textrm{V} O_2}$) and carbon dioxide output ($\dot{\textrm{V} CO_2}$) rates were collected using a portable gas exchange system (K5, COSMED, Italy). Kinematics of the lower body and torso were measured at 100 Hz using an inertial motion capture system (Xsens MVN Awinda, Movella, USA) with 8 inertial measurement units (IMUs) attached to the body. The IMUs were placed according to the manufacturer’s recommendations; i.e., near the sternum, on top of the sacrum, on the lateral flat surface of each thigh, on the flat surface of the shin bones right below each knee, and on each foot above the metatarsals. The foot IMUs were placed on top of the shoe and tightly secured with duct tape, and the rest of the IMUs were attached to the body using elastic straps holding the sensors tightly in place (see Fig. 1b).

#### Participants

Twelve able-bodied participants (4 women, 8 men; age 23.7 ± 2.7 years; body mass 67.4± 7.7 kg; height 1.75 ± 0.08 m) were recruited for the experimental testing. None of the participants had any previous experience of assisted walking with a hip exoskeleton prior to our experiments. The experimental protocol was reviewed and approved by the human research ethics committee of the canton of Vaud (CER-VD) under project ID 2023–02305 (Subproject 1). Participants provided their written informed consent before the experiments, which were conducted in accordance with the Declaration of Helsinki. The person depicted in Fig. 1b is one of the authors, who has given explicit consent for the publication of the image.

#### Testing protocol

To evaluate the controller under challenging and realistic conditions, we designed a mixed-terrain route emphasizing physically demanding activities where assistance is most relevant. The route comprised an indoor section within a commercial building, followed by an outdoor section in the adjacent urban area (Fig. 1c). The indoor part began with 2 min of level-ground walking for warm-up. This was followed by 2 min of simulated stair climbing, in which the participants were instructed to ascend a descending escalator while trying to maintain the same position, similar to using a stair stepper. Participants then walked to the starting point of the outdoor section (around 20 m away from the escalator), and during this period they were allowed to walk slowly to recover. This short transition period ($< {45} \, {s}$) was excluded from the analysis. The outdoor segment began with a mostly flat pathway leading to a 13% negative ramp (24 m in length), going down to a pedestrian underpass. The underpass ended with a flight of stairs ascending to a sidewalk. This sidewalk had an upward gradient increasing from 5% to 11% over the first 100 m, and continued at a nearly constant slope afterwards. The duration of the entire outdoor section was 6 min, with slightly different covered distances among participants depending on their preferred speed.

A preparatory session was scheduled 1–3 days before the main experiment to familiarize participants with the exoskeleton’s assistance, establish their preferred walking pace pattern, and collect HR calibration data. This session comprised three stages: (1) initial familiarization to the exoskeleton assistance in the laboratory, (2) acclimation to mixed-terrain assisted walking in a public setting involving level ground and stairs, and (3) familiarization to and pretesting of the main experimental route (with exoskeleton assistance), during which their preferred walking speed pattern was established by recording the time to reach predefined landmarks. $\mathrm {HR_{min}}$ and $\mathrm {HR_{max}}$ (used in Eq. 4) were defined as the minimum and maximum HR values recorded during stages 2 and 3.

For the main experiment, participants completed the route under three conditions (No Exo, Exo Off, Exo On). The order of conditions was pseudorandomized to ensure firstly that the exoskeleton conditions (Exo Off and Exo On) followed each other for practical reasons, and secondly to have a balanced number of the possible permutations. For the pseudorandomization, a list of the possible permutations of the conditions was generated manually, which was sorted using a random number generator and assigned to the participants sequentially. Before the walking tests, participants stood quietly for 5 min to measure their resting metabolic rate. For all sections except Escalator, participants followed an experimenter who set the pace based on their previously determined preferred speeds. More details about the protocol are given in Supplementary Methods.

### Data processing and analysis

#### Preprocessing

The data for the seven sections of the experimental route were first separated based on durations, motion capture recordings, and GPS coordinates recorded by the metabolic system. Stride segmentation was performed based on heel-strikes, detected as the most prominent peak in vertical foot velocity occurring when horizontal foot velocity dropped below 30% of its maximum. The heel-strike detection algorithm was validated against foot contact information obtained from the insole sensors for all sections. Due to symmetry, only the data for the left leg was used. Several parts of data had to be discarded due to measurement issues or anomalies. For more details about the preprocessing, see Supplementary Methods.

#### Calculation of metrics

For assistive torque amplitudes and profiles, we directly used the values calculated by the controller. Exoskeleton mechanical power was calculated as the product of actuator torque (estimated from motor currents) and hip flexion/extension angular velocity. Positive values of torque and angular velocity corresponded to the direction of extension. All torques and powers were normalized by participant body mass.

The reference values of torque amplitude used to evaluate the adaptation were based on the peak extension torques in average biological hip joint moment profiles. These peak values were extracted from the moment profiles reported in the literature for various locomotor tasks^30,31^ and then linearly mapped to the range of assistance amplitudes used by the controller (0.19–0.3 $\hbox {N} \cdot \hbox {m/kg}$), as detailed in Supplementary Methods and reported in Supplementary Table S1.

HR response time was defined as the time difference between the change in locomotor activity and the moment when HR began to consistently rise/fall for longer than a heuristic threshold. The rising and falling duration thresholds were set to 12 s and 20 s respectively, determined by trial and error to avoid false positives from acute HR variations in the absence of a major change. The only instances of abrupt change in intensity where this metric could be calculated were the start and end of Escalator, and the start of Stairs. HR time constant was defined as the time needed to cover 63.2% of the total difference between the starting and near-steady-state HR values. The near-steady-state value was approximated as the mean HR during the last 10% of the period of sustained activity. We calculated the time constant only for the rising HR phase in Escalator, as it was the only sustained period of increased effort after an abrupt change.

All kinematics and spatiotemporal parameters were extracted from motion capture measurements, which included all leg joint angles and segment positions. Stride length was calculated based on the distance between the initial and final positions of the left foot (coincident with the left ankle’s center of rotation in the motion capture system’s output). For walking, only the distance in the horizontal plane was considered, but for stair ascent the 3D distance was taken. Foot clearance in walking strides was calculated as the difference between the maximum and minimum foot heights. In stair ascent, foot clearance was defined as the difference between the maximum foot height and its value at the end of the stride.

The respiratory exchange ratio (RER) calculated from gas exchange measurements exceeded 1.0 toward the end of the Escalator section for all participants, indicating a shift towards an increased reliance on anaerobic metabolism. As a consequence, indirect calorimetry could not be used to accurately estimate the metabolic rate over the entire route. We therefore directly used $\dot{\textrm{V}O_2}$ as the measure of physical effort intensity. Net $\dot{\textrm{V}O_2}$ was calculated by subtracting the average resting $\dot{\textrm{V}O_2}$ (calculated based on the last minute of the five minutes of quiet standing) from the gross $\dot{\textrm{V}O_2}$ values. The values were normalized by each participant’s body mass prior to analysis. To check for near-steady $\dot{\textrm{V}O_2}$ behavior in the last 30 s of Escalator and Incline cont., we used the common criterion of a variation coefficient below 10%^52^. As detailed in Supplementary Methods, this criterion was met in nearly all cases (34 out of 35 datasets for Escalator, and 33 out of 36 for Incline cont.).

#### Statistical analysis

To check for significant differences in means of net $\dot{\textrm{V}O_2}$, kinematic and spatiotemporal metrics, a linear mixed-effects model was used, which allows retaining participants with partly missing data. Therefore, only the missing values were excluded from the analysis. The conditions (No Exo/Exo Off/Exo On) were set as fixed effects, and participants as a random effect to account for the repeated measures. The normality of the residuals was tested and confirmed using the Kolmogorov-Smirnov test. Post-hoc comparisons were carried out with the Holm correction to take the repeated pairwise comparisons into account. The level of significance for all comparisons was $\alpha = 0.05$. As a measure of effect size for the changes in $\dot{\textrm{V}O_2}$, we calculated Cohen’s *d* for paired samples, where scores below 0.35 can be interpreted as trivial, 0.35 to 0.80 as small, 0.80 to 1.50 as moderate, and greater than 1.5 as large effects^53^. To analyze the correlation between the assistance torque amplitudes and the biological reference values, we calculated Pearson’s product-moment coefficient, using the threshold of $r> 0.7$ to indicate a strong correlation, which is commonly accepted in biostatistics^54^. Statistical analysis was conducted in Jamovi software version 2.3^55,56 ^for all analyses, except for Pearson’s correlation analysis, which was carried out in MATLAB R2022b^28^.

## Supplementary Information

Supplementary Information 1.

Supplementary Information 2.

## Data Availability

The collected raw data and the compiled data for analysis in the current study are available in the Zenodo repository, accessible at https://doi.org/10.5281/zenodo.11357721.
