# Supplementary material for: Adaptive hip exoskeleton control using heart rate feedback reduces oxygen cost during ecological locomotion
Source: Sci Rep. 2025 Jan 2;15:507. doi: 10.1038/s41598-024-84253-y (PMC11695581; doi:10.1038/s41598-024-84253-y)
Supplement: Supplementary file 1 — Supplementary Information 1. [file 41598_2024_84253_MOESM1_ESM.pdf]

Supplementary Material for:

Adaptive Hip Exoskeleton Control using Heart Rate Feedback Reduces Oxygen Cost during Ecological Locomotion

Ali Reza Manzoori, Davide Malatesta, Alexandre Mortier, Johan Garcia, Auke Ijspeert, and Mohamed Bouri

1 Supplementary Methods

1.1 Hip exoskeleton

The e-Walk V2 hip exoskeleton (Fig. S1) used in the experiments is a research prototype developed in our laboratory. The actuators are DC motors with a 6:1 planetary reducer, capable of nominal and peak torques of 13 and 35 $\mathrm{N \cdot m}$ at the output. In zero-torque mode, the RMS backdriving torque of the actuators is limited to 0.6 $\mathrm{N \cdot m}$ at frequencies of up to 2 Hz. Passive hinge joints connect the actuators to the exoskeleton's frame, allowing free hip abduction/adduction movement. A commercial orthosis was used for the thigh cuffs, which are connected to the exoskeleton's thigh segments using spherical joints enabling hip internal/external rotation as well. The main frame of the exoskeleton consists of a rigid external shell made of hard plastic placed around the lower back and the hips, with a soft lumbar brace on the inside for attachment to the user's waist. The width of the rigid shell and the relative height of the motors with respect to it are adjustable to fit the user's body. The thigh segments are also made of hard plastic, with a prismatic joint at the end for leg length adjustability and improved tolerance of slight misalignments between the exoskeleton and the user's hip joints.

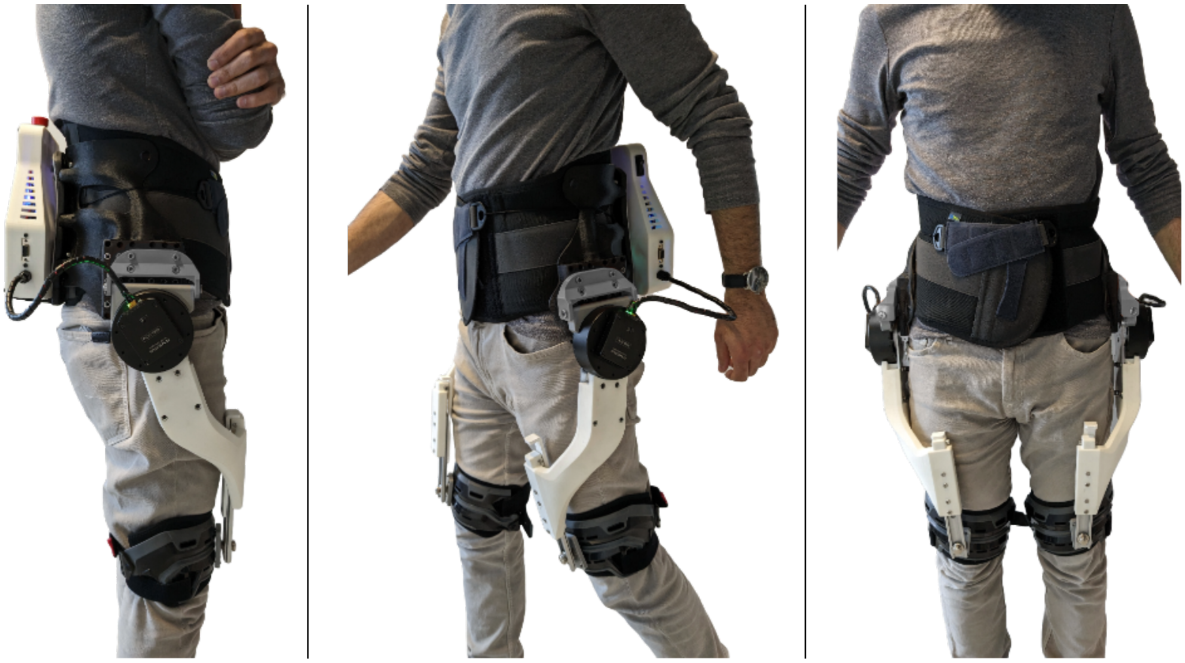

Figure S1. The e-Walk V2 hip exoskeleton.

The exoskeleton torques were estimated from the currents measured by motor drivers, based on a linear model previously identified in benchtop experiments with a torque sensor. The inverse model was also used to convert the torque commands generated by the controller to current commands sent to the drivers, which regulate the motor current at 32 kHz. Approximate vertical ground reaction forces used in the controller were measured using insole force-sensitive resistors (8-cell Smart Foot Sensor, IEE, Luxembourg), sampled with custom amplifier boards placed around the ankles and connected to the electronics enclosure of the exoskeleton via a cable (Fig. S2). The measured resistance of each individual cell was converted to an

approximate force using a linear model. The coefficients of the model were determined by a two-point calibration with 0 N and 10 N external forces. Note that since only the ratio of the ground reaction forces was required for the controller (as described in Eq. 1), an accurate calibration of the forces was not necessary. All peripherals (motor drivers, insole sensor amplifiers, and the heart rate sensor) directly communicate with the exoskeleton's embedded computer (BeagleBone Black, BeagleBoard.org Foundation, USA). The computer logged the data and ran the controller at a frequency of 500 Hz.

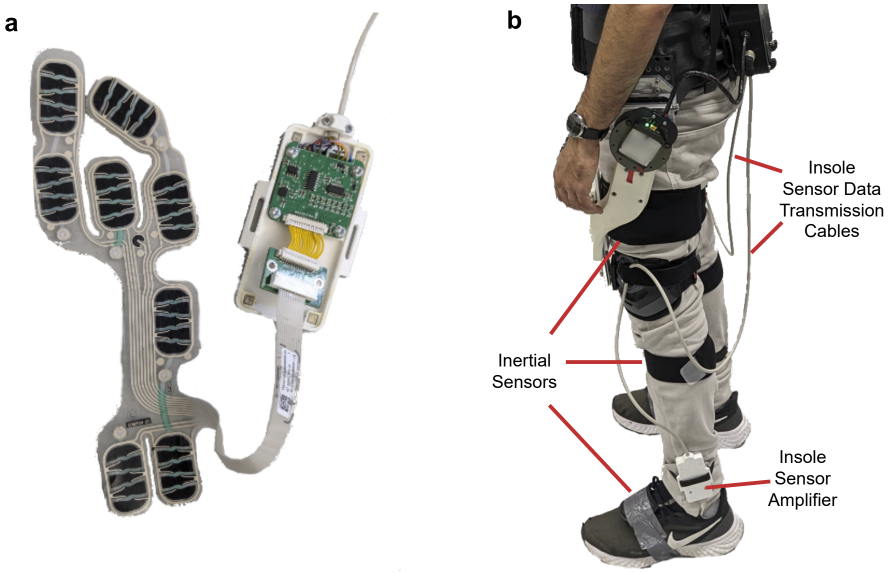

Figure S2. The sensing and amplification setup for vertical ground reaction force sensing using force-sensitive resistors. **(a)** The insole force-sensitive resistor set and the amplification board. The sensors were placed inside of the shoes and covered with thin shoe inserts for protection. **(b)** Placement of the amplifier box around the user's ankle and its cable connection to the exoskeleton's electronics enclosure.

1.2 Experimental procedure

1.2.1 Details of the testing route

The experimental route was conceived to cover a variety of terrains typically encountered in daily living in an interrupted scenario, with more emphasis on physically demanding ones. This consisted of walking on level and inclined ground and ascending stairs. Stair descent was excluded due to its lower energetic demands and the predominantly eccentric hip action, where active exoskeleton assistance is less relevant. The various sections of the route are shown in the Supplementary Video.

As explained in the main article, we fixed the durations (2 min each for Indoor flat and Escalator, and 6 min for the entire outdoor part). Therefore, the covered distance for each participant depended on their walking speed. For Indoor flat, we calculated the distance to be traveled for each participant based on their preferred speed, which was around 132 m on average. The escalator used for simulated stair ascent had a step height of 20 cm and a rate of 1.2 steps/s. The length of the Outdoor init. section was approximately 130 m, and the Underpass was around 50 m long (including the entrance ramp). The flight of stairs at the end of the underpass (Stairs) consisted of 24 steps with a height of 17 cm. The Incline init. section was 90 m long, and the maximum distance covered in Incline cont. was 212 m. The elevation profile for the outdoor section of the route (calculated using the barometric altitude measured by the respirometry system) is illustrated in Fig. S3. Note that most of the participants did not cover the entire 500 m shown in this figure within the allotted 6 min.

1.2.2 Familiarization

For basic familiarization, the participants were first asked to walk with exoskeleton assistance in the laboratory, while we gradually increased the assistance amplitude. This phase continued until the participant verbally indicated feeling confident while walking with the assistance. Then, they were asked to walk along a public route consisting of various terrains (level ground, inclined ground, and stairs) for about 10 min with a fixed level of assistance. This route led to the starting point of the outdoor section of the main experimental route. The participants were then instructed to follow the outdoor section at their comfortable pace while assisted with the exoskeleton. They were free to change their pace naturally according to the terrain. During this phase, we recorded the elapsed times at pre-determined landmarks along the route to use as targets for maintaining a consistent walking speed across the conditions during the main experiment. It was not possible to include the indoor section

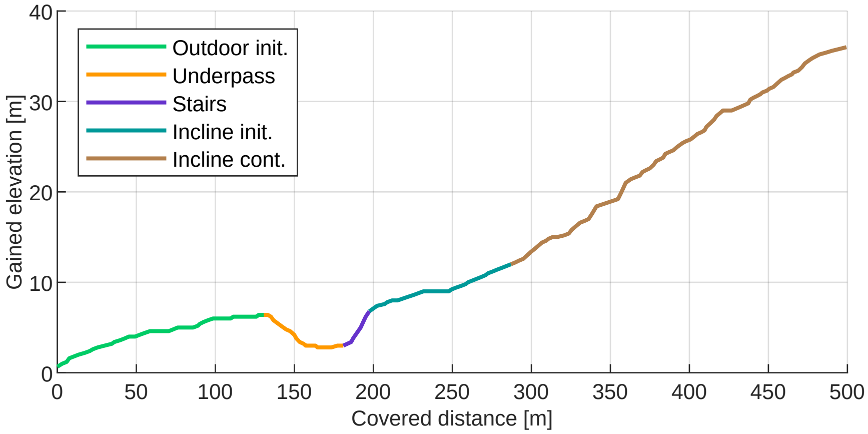

Figure S3. The elevation profile of the outdoor part of the experimental route, using the starting point of the route as the elevation reference.

in the familiarization since access to the commercial building was restricted to a limited duration, which was sufficient for the final measurements only. Therefore, the average walking speed recorded in the flat part of the pedestrian underpass during familiarization was used as the preferred speed for the "Indoor flat" section as well. In the "Escalator" section of the indoor part, the stepping speed was enforced by the escalator.

1.2.3 Data collection

Inertial motion capture. For the kinematic measurements, we used the "Lower Body with Sternum" configuration of the Xsens MVN Awinda system, in the "Multi Level" recording scenario (which is intended for measurements involving major changes in height, such as stair climbing). The IMUs were placed according to the manufacturer's instructions. Three sensors were placed on each leg (on the thigh, shank, and foot segments), in addition to one sensor on the lower back and another one on the chest near the sternum. The standard calibration procedure was carried out once prior to the conditions with the exoskeleton (Exo Off and Exo On), and once before the No Exo condition, to account for possible small sensor displacements due to donning and doffing of the exoskeleton. In this procedure, the participant is first asked to stand still for a few seconds in a known posture (referred to as "N-Pose" by the manufacturer[1]), and then walk around slowly until the software indicates the collection of sufficient calibration data. In cases where the initial calibration quality was deemed unsatisfactory by the software, we repeated the calibration.

Respirometry. The gas exchange measurement system was calibrated once prior to data collection for each participant. The calibration consisted of an initial step with ambient air, a flowmeter calibration, zeroing CO_2 using a scrubber, and calibration with a reference gas. Participants were asked to fast (except for drinking water) in the 3 h prior to the experiment. In previous measurements during our earlier study[2], we did not observe significant differences in the resting gas exchange rate between measurements taken with and without wearing the exoskeleton. Therefore, the 5 min of quiet standing measurement was taken while wearing the exoskeleton for participants starting the experiment with Exo Off or Exo On, and without the exoskeleton for those commencing with No Exo.

Synchronization. The data recorded by all systems (i.e., the exoskeleton, the inertial motion capture system, and the gas exchange measurement system) were synchronized during the collection procedure. To synchronize the exoskeleton and the motion capture system recordings, a digital pulse was sent from the motion capture system to the exoskeleton to mark the start of recording. The gas exchange measurements were also manually initiated at the same time.

Ambient conditions. The main experiments were carried out on four consecutive days, at the same period of the day (morning to noon). The ambient temperature, relative humidity, and pressure were measured with the built-in sensors of the respirometry system. We also measured wind speeds near the end of the route (where the altitude was higher and measurable winds were more likely to be present) using an anemometer (Vortex, Inspeed LLC, USA). In the indoor part, ambient temperatures were between 24 and 26 °C, relative humidity was between 50 and 65 %, and ambient pressures were in the 730–731 mmHg range. In the outdoor part, temperatures ranged from 24 to 32 °C, relative humidity was between 37 and 62 %, and ambient pressures were around 727–730 mmHg. There were no strong winds, and the maximum measured wind speed was 1.8 m/s.

1.3 Preprocessing

Motion capture reprocessing. Inertial motion capture data were preprocessed using the manufacturer's standard software ("HD reprocessing" in Xsens MVN Analyze, Version 2023.2.0), which utilizes non-causal filtering using a larger window[3] to improve recording quality prior to the final analysis.

Measurement issues. During the initial inspection, we noticed issues in the data and therefore several parts were discarded from the analysis. The Escalator section of the Exo On condition for Participant 1 had to be excluded from the analysis, since the participant stayed too close to the landing platform of the escalator where the steps did not reach their full height. Furthermore, there were several instances of missing (due to software failure) or unusable (due to too many lost packets, as determined by the motion capture software) motion capture data. For a few participants and conditions, data for some sections were entirely unavailable or discarded. This included the entire Exo Off condition for Participant 1, Escalator and Incline cont. sections in Exo On for Participant 2, the entire Exo On condition for Participant 4, both indoor sections in Exo Off for Participant 8 and in Exo On for Participant 9, and the Indoor flat section in Exo Off for Participants 10 and 12.

Data segmentation. For segmenting the data into the sections, we used the GPS coordinates (which were recorded by the respirometry system) for all outdoor sections, except the transition from Underpass to Stairs for which the GPS data was not reliable in the covered pedestrian underpass. We therefore used the motion capture recordings to detect the start of stair climbing in this section. For the indoor sections where GPS data was unavailable, we detected the start of measurements from the motion capture data, and then segmented the two sections based on their fixed duration of 2 min. We also validated the time-based segmentation against motion capture recordings when available, which showed good agreement (temporal errors below 5 s).

1.4 Analysis

Calculation of the biological reference torque amplitudes. To calculate the biological reference torque amplitudes for each section, we used the hip moment profiles reported in the literature for similar terrain types and gait speeds. Profiles were sourced from the dataset published by Camargo et al.[4], except for the Escalator section, where we used data from McCabe et al.[5] due to the closer match in conditions (stair climber machine rather than regular stairs, with similar step heights to those of our escalator). The locomotion task used as reference for each section is described in Table S1. Note that for Incline init. and Incline cont., we used linear extrapolation or interpolation, since the ramp angles of these sections did not exist in the reference dataset. We defined the biological torque amplitude for each task as the maximum extension torque in the across-participants average profile. For better comparison with the exoskeleton torques, we linearly scaled the values to the range of torque amplitudes used by the controller. The scaling was defined so that the biological amplitude at the slowest gait speed observed during familiarization (corresponding to the lower bound of the heart rate, HR_{min} in Eq. 4) was mapped to the lowest exoskeleton torque amplitude ($0.19\,\mathrm{N \cdot m/kg}$), while the biological amplitude corresponding to the most demanding activity was mapped to the highest exoskeleton torque ($0.3\,\mathrm{N \cdot m/kg}$). The raw and scaled biological torque values are also reported in Table S1.

Checking for near-steady $\dot{V}O_2$ behavior. To verify whether $\dot{V}O_2$ reached a nearly stable level in the last 30 s of the Escalator and Incline cont. sections, we first smoothed the signal by calculating a 5-sample moving average. Then we calculated the coefficient of variation (CV) of the signal over the entire duration of the section using a 30-second moving window and compared the values against a threshold of 10% (CV < 0.1). For Escalator, in only 1 out of the 35 valid datasets (Participant 10 in the Exo On condition) CV briefly reached 10.3%, while in the majority of the datasets (31/35) its value remained near or below 5%. For Incline cont., in 3 out of 36 datasets CV did not fall below 10% during the last 30 s (Participant 2 in No Exo: up to 11.6%, Participant 10 in Exo Off: up to 10.7%, and Participant 12 in Exo On: up to 17%). Again, in most of the remaining cases (28/36), CV was near or below 5%.

Current consumption and battery life analysis. To quantify the impact of a constantly high assistance on the exoskeleton's operational time, we compared the motor current in our experiments (adaptive scenario) with a hypothetical case in which the amplitude was fixed at $\tau_{max} = 0.3\,\mathrm{N \cdot m/kg}$ (fixed scenario). For this comparison, we used the quadrature motor currents (the effective current proportional to BLDC motor torque) recorded during the experiment. We used the data for Participant 11, whose body mass (69 kg) was near the average of our sample. For the adaptive scenario, we averaged the total current of both left and right motors over the entire duration of the experiment. To estimate the currents for the fixed scenario, we first divided the recorded currents by the actual instantaneous torque amplitude recorded in the experiment (τ_A), scaled the result by τ_{max}, and then calculated the average over the entire duration. The resulting average currents were 13.88 A (adaptive), versus 16.53 A (fixed). Note that this 19% increase of the effective current in the fixed scenario would translate to larger increases in the total consumed current, since losses scale up non-linearly with current and torque.

Locomotion Task	Raw Amplitude [N · m/kg]	Scaled Amplitude [N · m/kg]	Use Case
Overground walking, 0.8–1.0 m/s[4]	0.380	0.190	Lower bound of the raw amplitudes
Overground walking, 1.0–1.2 m/s[4]	0.400	0.193	Reference for Indoor init.
Ascending stair climber, step height 203 mm[5]	1.060	0.300	Reference for Escalator and upper bound of the raw amplitudes
Transition from ramp ascent (5.2°) to level-ground walking[4]	0.580	0.222	Reference for Outdoor init.
Transition from ramp descent (−7.8°) to level-ground walking[4]	0.410	0.195	Reference for Underpass
Ascending stairs, step height 178 mm[4]	0.680	0.239	Reference for Stairs
Ascending 3.9° ramp[4] (extrapolated from reported ramp values)	0.620	0.229	Reference for Incline init.
Ascending 6.5° ramp[4] (interpolated from reported ramp values)	0.830	0.263	Reference for Incline cont.

Table S1. Summary of the raw and scaled biological torque amplitudes used for validating the controller's amplitude adaptation. The scaling was defined so as to map the range of biological torque amplitudes to the range of assistive torque amplitudes used in the controller. The first column describes the corresponding locomotion task in the reference dataset, and the last column specifies the use case of each value, either as the reference for a section or as a bound to define the scaling.

2 Supplementary Results

2.1 Heart rate response times

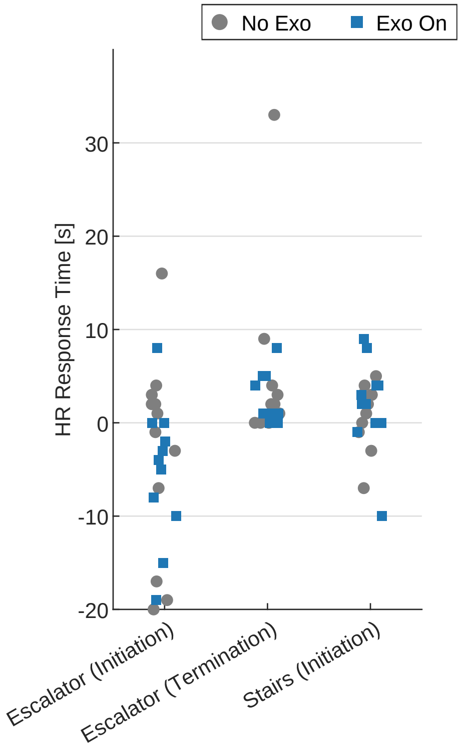

Figure S4. The heart rate response times for the individual participants under both No Exo and Exo On conditions.

2.2 Joint kinematics profiles

The mean angle profiles for hip, knee and ankle joints in the sagittal plane are shown for the 7 sections in Figs. S5–S11. To obtain these profiles, the average profile for each participant was first calculated and then the overall mean was calculated from the individual average profiles.

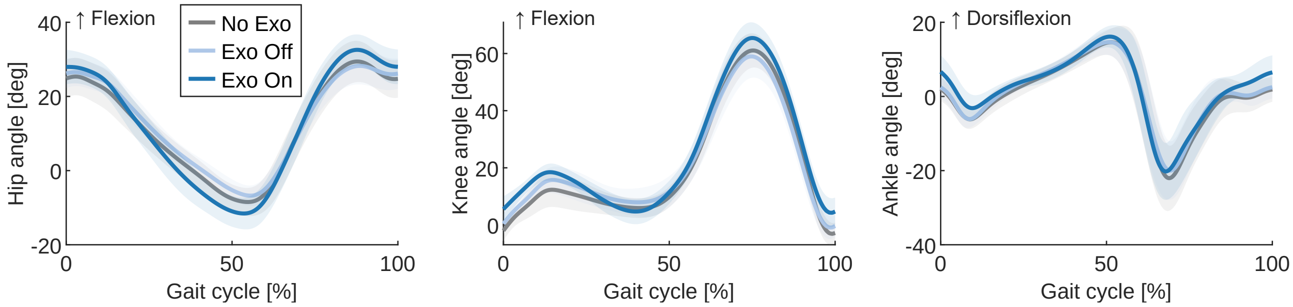

Figure S5. The average sagittal joint angle profiles calculated over all participants with valid measurements in each condition for Indoor flat ($n_{\text{NoExo}} = 12$, $n_{\text{ExoOff}} = 8$, $n_{\text{ExoOn}} = 10$). The shaded areas mark the standard deviation for each profile.

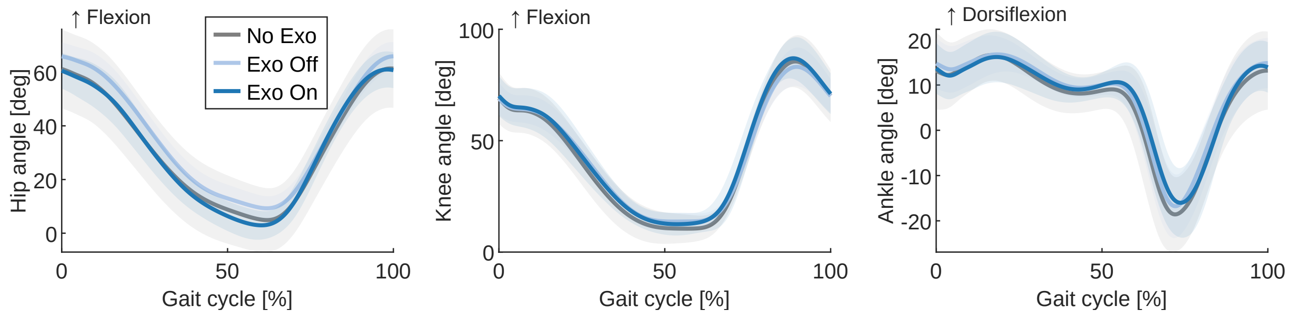

Figure S6. The average sagittal joint angle profiles calculated over all participants in each condition for Escalator ($n_{\text{NoExo}} = 11$, $n_{\text{ExoOff}} = 10$, $n_{\text{ExoOn}} = 10$). The shaded areas mark the standard deviation for each profile.

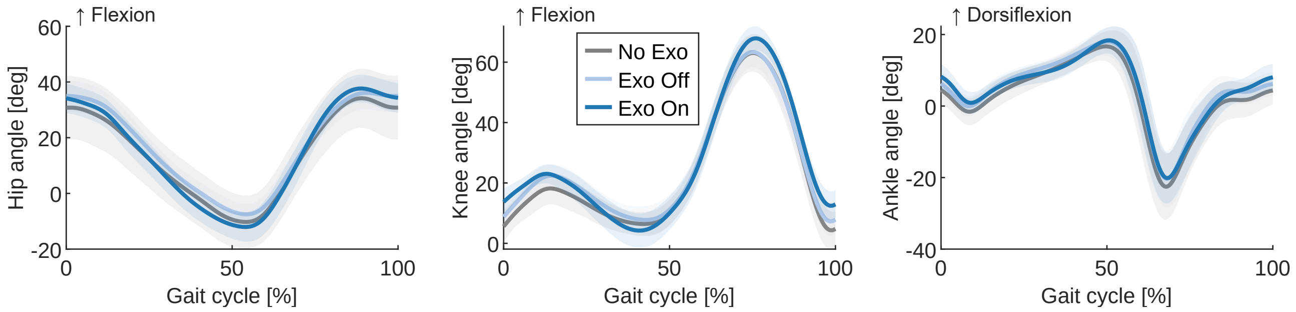

Figure S7. The average sagittal joint angle profiles calculated over all participants in each condition for Outdoor init. ($n_{\text{NoExo}} = 12$, $n_{\text{ExoOff}} = 11$, $n_{\text{ExoOn}} = 11$). The shaded areas mark the standard deviation for each profile.

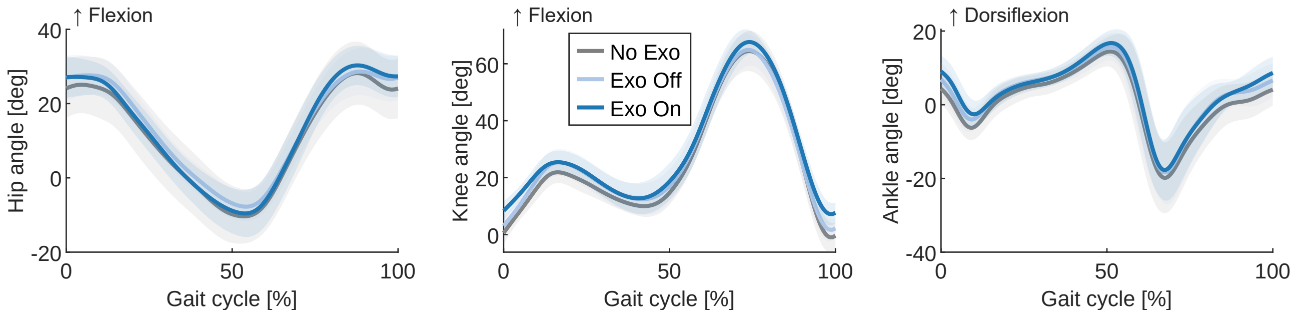

Figure S8. The average sagittal joint angle profiles calculated over all participants in each condition for Underpass ($n_{\text{NoExo}} = 12$, $n_{\text{ExoOff}} = 11$, $n_{\text{ExoOn}} = 11$). The shaded areas mark the standard deviation for each profile.

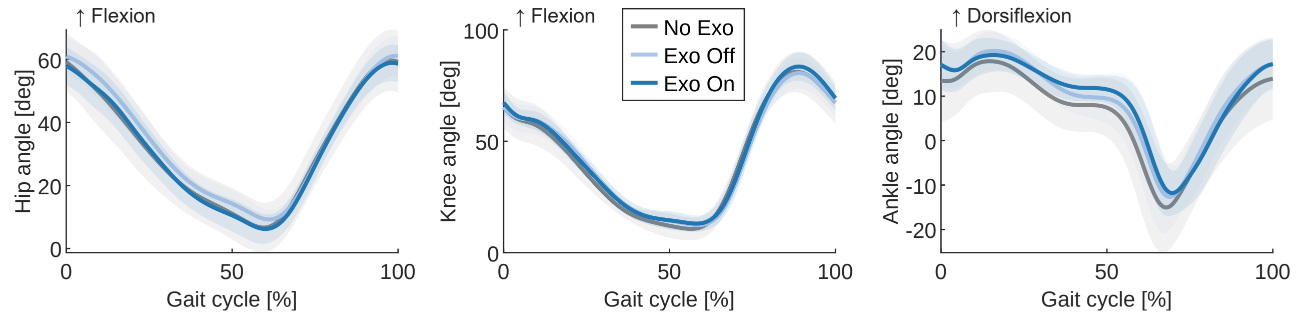

Figure S9. The average sagittal joint angle profiles calculated over all participants in each condition for Stairs ($n_{\text{NoExo}} = 12$, $n_{\text{ExoOff}} = 11$, $n_{\text{ExoOn}} = 11$). The shaded areas mark the standard deviation for each profile.

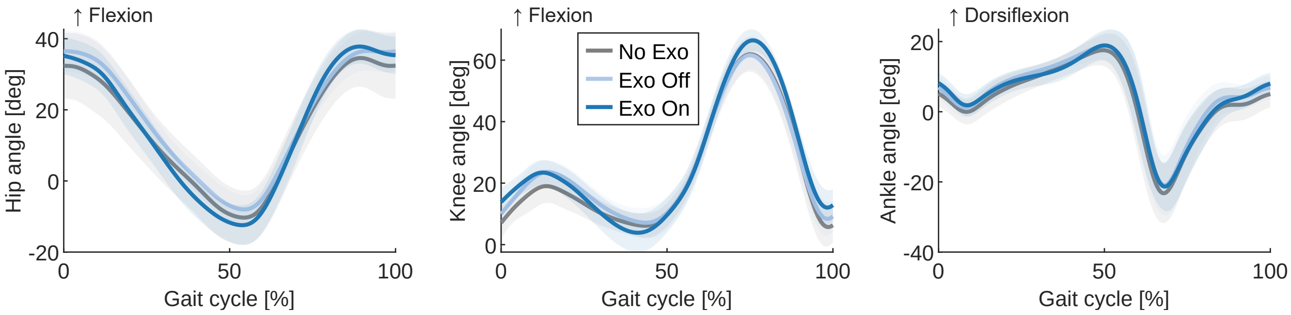

Figure S10. The average sagittal joint angle profiles calculated over all participants in each condition for Incline init. ($n_{\text{NoExo}} = 12$, $n_{\text{ExoOff}} = 11$, $n_{\text{ExoOn}} = 11$). The shaded areas mark the standard deviation for each profile.

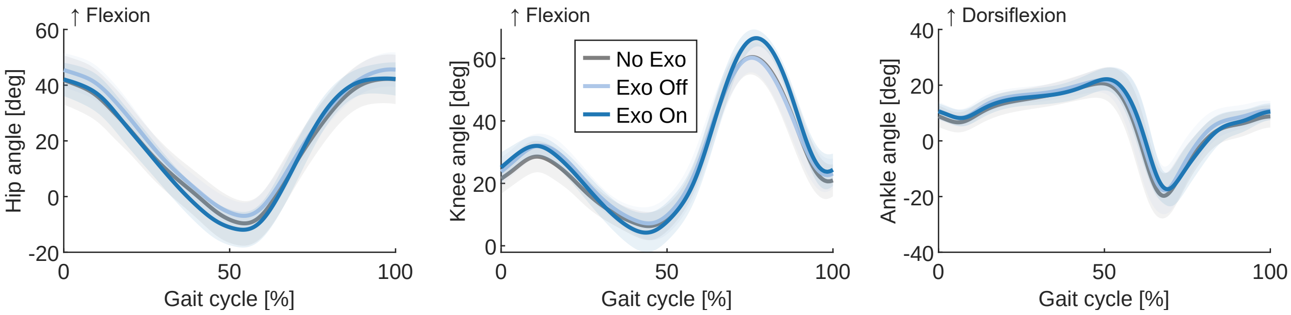

Figure S11. The average sagittal joint angle profiles calculated over all participants in each condition for Incline cont. ($n_{\text{NoExo}} = 12$, $n_{\text{ExoOff}} = 11$, $n_{\text{ExoOn}} = 10$). The shaded areas mark the standard deviation for each profile.

3 Supplementary Video

The supplementary video includes brief footage snippets showing the various sections of the experimental route.

References

1. Schepers, M., Giuberti, M. & Bellusci, G. Xsens MVN: Consistent Tracking of Human Motion Using Inertial Sensing. Technical Report, XSENS TECHNOLOGIES B.V. (2018). DOI: 10.13140/RG.2.2.22099.07205.
2. Manzoori, A. R., Malatesta, D., Primavesi, J., Ijspeert, A. & Bouri, M. Evaluation of controllers for augmentative hip exoskeletons and their effects on metabolic cost of walking: explicit versus implicit synchronization. *Front. Bioeng. Biotechnol.* **12**, DOI: 10.3389/fbioe.2024.1324587 (2024).
3. Movella. Reprocess (HD) (2023). URL: https://base.movella.com/s/article/Reprocess-HD?language=en_US.
4. Camargo, J., Ramanathan, A., Flanagan, W. & Young, A. A comprehensive, open-source dataset of lower limb biomechanics in multiple conditions of stairs, ramps, and level-ground ambulation and transitions. *J. Biomech.* **119**, 110320, DOI: 10.1016/j.jbiomech.2021.110320 (2021).
5. McCabe, M. V., Van Citters, D. W. & Chapman, R. M. Hip Joint Angles and Moments during Stair Ascent Using Neural Networks and Wearable Sensors. *Bioengineering* **10**, 784, DOI: 10.3390/bioengineering10070784 (2023).
